# Toothpick perforates small bowel-mimicking diverticulitis

**DOI:** 10.1093/jscr/rjad511

**Published:** 2023-09-18

**Authors:** Ahmed A Fallatah, Hassan Mashbari, Hatoon Daghestani, Omar Mostafa, Nawaf Alshahwan, Ahmed Alburakan, Thamer Nouh

**Affiliations:** Trauma and Acute Care Surgery Unit, Department of Surgery, College of Medicine, King Saud University, P.O Box 2925 Riyadh 11461, Saudi Arabia; General Surgery Section, Department of Surgery, King Abdulaziz Hospital, Ministry of Health, P.O Box 3665 Jeddah 22421, Saudi Arabia; Department of Surgery, Faculty of Medicine, Jazan University, P.O Box 6809 Jazan 82817-28204, Saudi Arabia; Trauma and Acute Care Surgery Unit, Department of Surgery, College of Medicine, King Saud University, P.O Box 2925 Riyadh 11461, Saudi Arabia; College of Medicine, Sulaiman Al Rajhi University, P.O Box 777 Albukairiyah 51941, Saudi Arabia; Trauma and Acute Care Surgery Unit, Department of Surgery, College of Medicine, King Saud University, P.O Box 2925 Riyadh 11461, Saudi Arabia; Trauma and Acute Care Surgery Unit, Department of Surgery, College of Medicine, King Saud University, P.O Box 2925 Riyadh 11461, Saudi Arabia; Trauma and Acute Care Surgery Unit, Department of Surgery, College of Medicine, King Saud University, P.O Box 2925 Riyadh 11461, Saudi Arabia

**Keywords:** toothpick, perforation, foreign body, small bowel, CT scan, abdominal pain

## Abstract

Bowel perforation is an emergency condition that requires critical thinking and readily intervention; nevertheless, on occasions, its presentation can be challenging to diagnose. Several etiologies could cause bowel perforation, including obstruction, mass, inflammation, ischemia, etc. On rare occasions, a foreign body could be the cause of perforation, which mandates a detailed history and focused review of the images when the patient’s condition allows. We report a case of ileal perforation caused by an ingested wooden toothpick that was suspected on the CT images, which the patient has no memory of ingesting.

## Introduction

Foreign body (FB) ingestion is a common emergency presentation, with a higher incidence observed in the pediatric and geriatric age groups. While most cases of FB ingestion pass through the gastrointestinal (GI) tract without causing any noticeable symptoms, there are situations where it becomes a surgical emergency and requires intervention. Bowel perforation, although rare, can occur in <1% of cases, with sharp objects being the main culprits in such instances.

The diagnosis of FB ingestion can be challenging, especially when the object is not radiopaque or when it becomes surrounded by inflammatory processes that hinder visualization on imaging studies. This can make it difficult to determine the presence and location of the FB. In such cases, diagnostic laparoscopy is an excellent surgical option. It allows for the direct visualization of the GI tract and the identification of the etiology behind the patient’s symptoms. This approach enables surgeons to accurately diagnose and manage the condition, offering a more targeted and effective treatment plan. If an FB is identified during the procedure, surgical intervention can be performed to remove it. Laparoscopy offers several advantages over open surgery, including smaller incisions, reduced postoperative pain, shorter hospital stays, and faster recovery times.

In this case report, we present a unique and uncommon occurrence of FB ingestion that posed a diagnostic challenge but was ultimately managed successfully after careful evaluation and consideration.

## Case report

A 36-year-old female, not known to have a medical illness, underwent laparoscopic Sleeve Gastrectomy 3 years ago and laparoscopic cholecystectomy 1 year ago. She was presented to the emergency department on 2 June 2023 with a 1-day history of sudden, severe, left-lower-quadrant (LLQ) pain that was nonradiating, scaled 7/10, and associated with nausea and vomiting. She denies a history of diarrhea, constipation, fever, melena, or hematochezia. On clinical examination, the patient’s vitals were within normal ranges.

**Figure 1:**
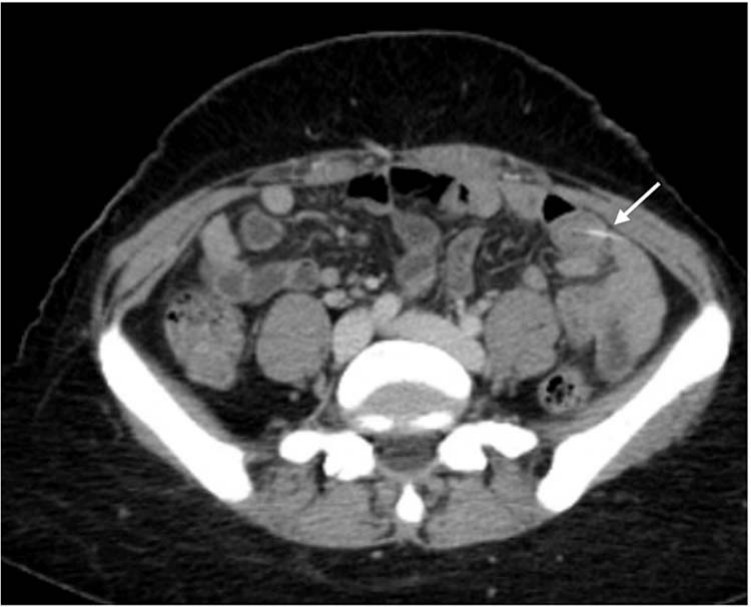
Axial views of contrast-enhanced CT of the abdomen showed a transversely oriented linear hyperdense FB (arrow) structure seen within the ilea loops in the lower quadrant.

**Figure 2:**
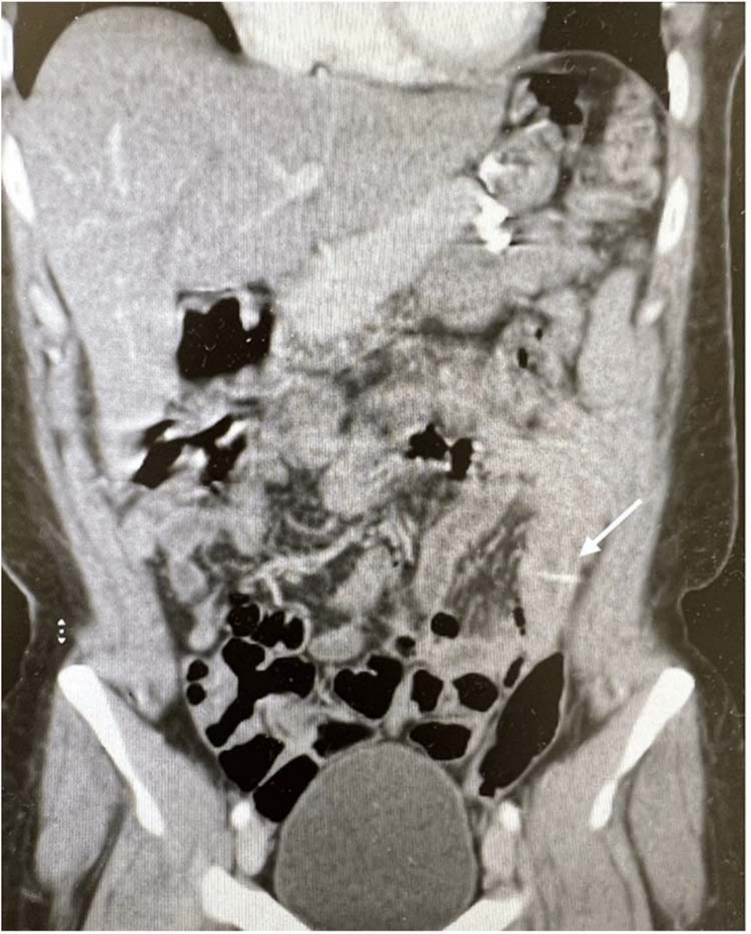
Coronal views of contrast-enhanced CT abdomen showed: a transversely oriented linear hyperdense FB (arrow) structure seen within the ilea loops in the lower quadrant.

The abdominal exam showed severe tenderness in LLQ + rebound tenderness. The white blood cell count was 9000/mm^3^. Contrast-enhanced CT of the abdomen (Figs 1 and 2) showed a transversely oriented linear hyperdense FB structure seen within the ilea loops in the LLQ measuring ~2 mm in thickness and 18 mm in length traversing the bowel wall, which demonstrates localized mural wall edema with minimal surrounding fatty haziness; however, there was no adjacent extra-luminal air or localized collections, and right adnexal cyst.

More history was obtained from the patient, specifically focusing on FB ingestion; she denied intentional ingestion of FBs or eating fish any time before the initiation of her symptoms. Obstetrics and gynecology service was consulted regarding the right adnexal mass on CT in the same admission, and ultrasound was performed and it showed neither adnexal mass nor ovarian abnormalities.

**Figure 3:**
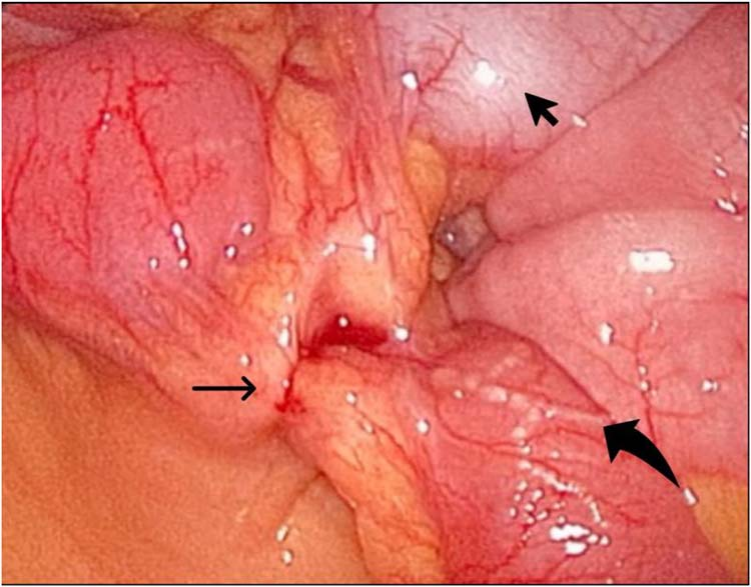
The laparoscopic exploration showed the sigmoid colon attached to the area of the inflammatory reaction; straight arrow: site of the toothpick; curved arrow: site of toothpick erosion through the bowel wall; arrowhead: sigmoid colon.

**Figure 4:**
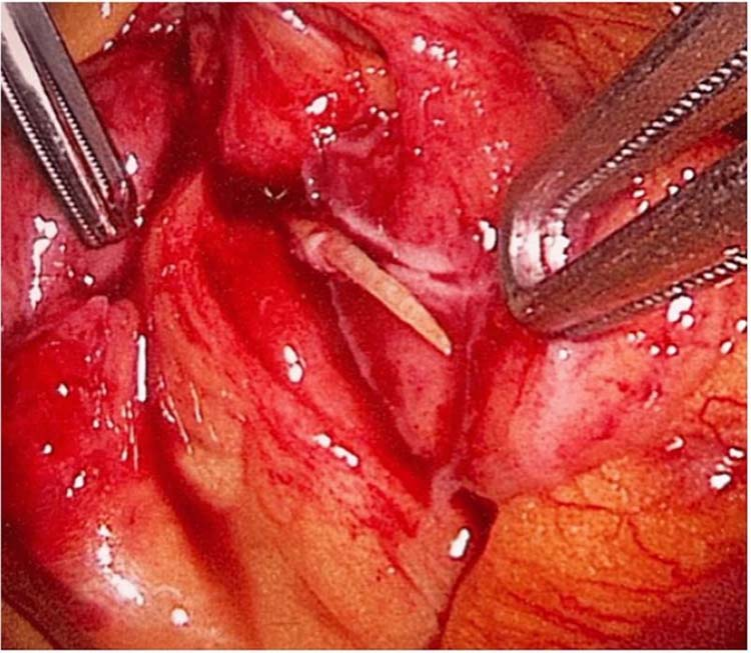
The laparoscopic exploration showed a FB in the mesentery of two loops of the ileum 100 cm from the ileocecal junction—upon partial exposure of the site of the toothpick.

**Figure 5:**
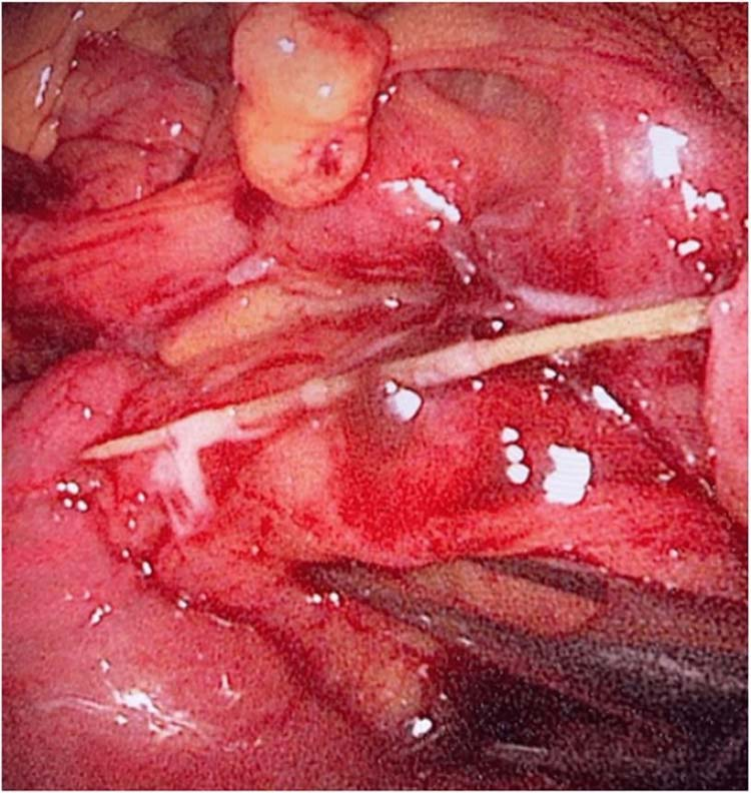
The laparoscopic exploration showed a FB in the mesentery of two loops of the ileum 100 cm from the ileocecal junction—after full exposure of the site of the toothpick*.*

**Figure 6:**
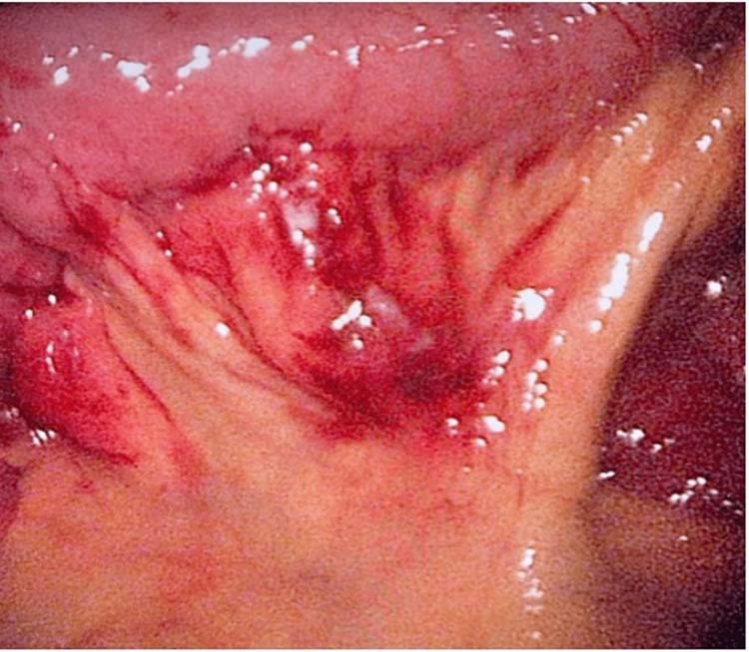
Site of the toothpick after removal.

**Figure 7:**
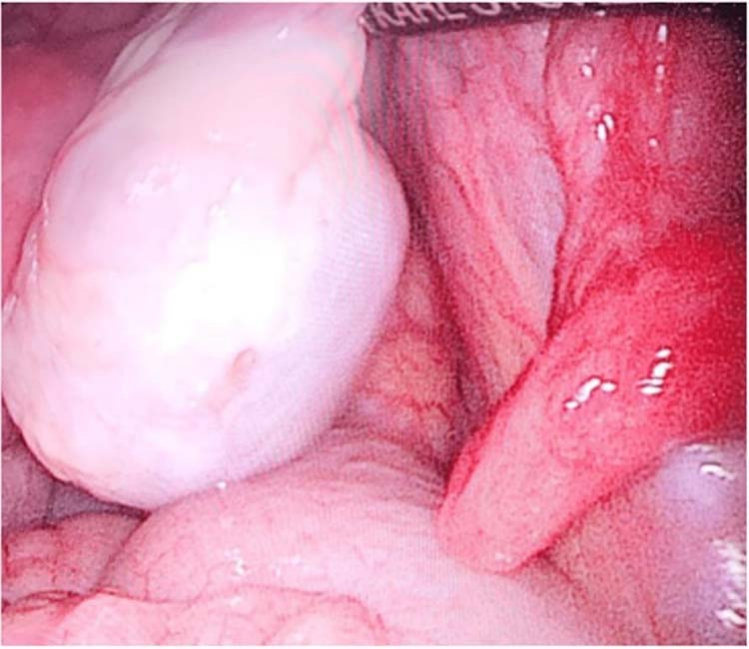
Right ovary.

**Figure 8:**
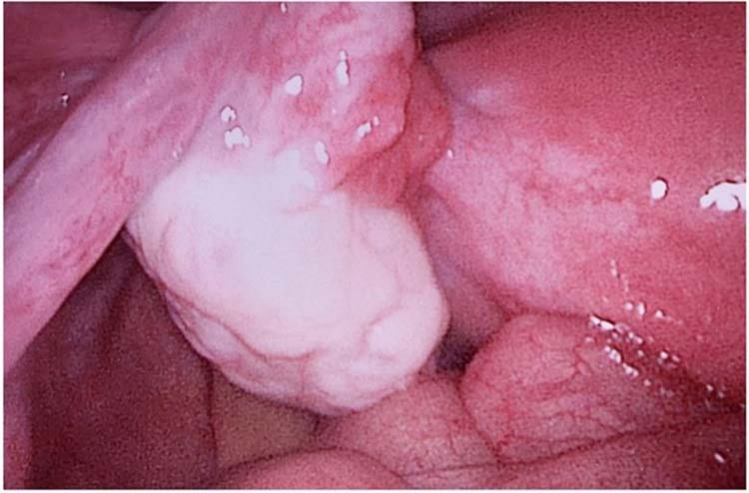
Left ovary.

**Figure 9:**
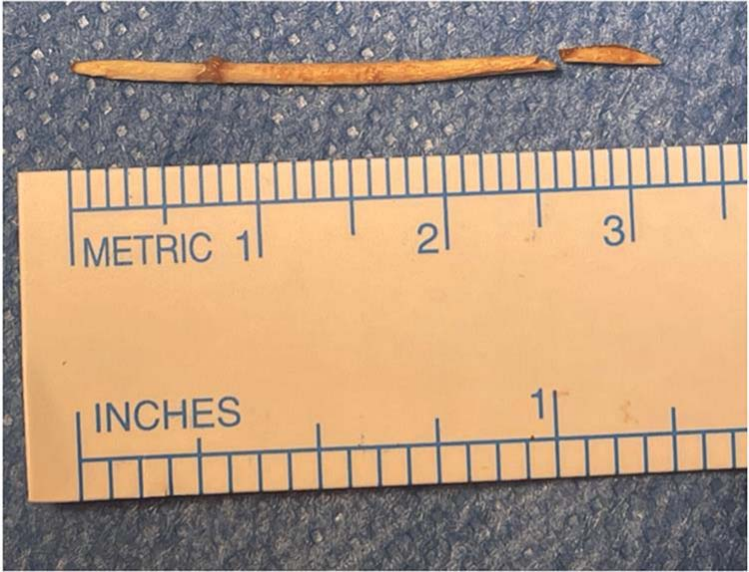
The FB was identified as a toothpick, 3 cm in length.

During her hospital stay, the patient showed no improvement regarding abdominal pain, nausea, or abdominal exam. Therefore, she was offered a laparoscopic exploration to verify the diagnosis of a FB to which she agreed. She was taken to the operating room, and the exploration showed an inflammatory process in the LLQ (Fig. 3) with the sigmoid colon attached to the area of the inflammatory reaction. A FB was found in the mesentery of two loops of the ileum 100 cm from the ileocecal junction (Figs 4 and 5). Removal of the FB safely and milking of the small bowel showed no leakage of bowel contents (Fig. 6). And, exploring the rest of the abdomen showed no other pathology and normal ovaries (Figs 7 and 8); the procedure was uneventful. The FB was identified as a toothpick, 3 cm in length (Fig. 9).

The patient recovered without complications and was discharged home on Postoperative Day 2. Upon follow-up in the clinic, the patient reported the resolution of all her symptoms and was discharged from the clinic.

## Discussion

In this rare presentation of ingested FB, the diagnosis was quite challenging, given the fact that most of the patients in their full state of mind deny ingestion of FB, as its ingestion in this age group is improbable, and possibly an adult individual would memorize or notice ingesting a FB.

FB ingestion is an emergency condition that requires prompt attention. The most affected populations are the pediatric, geriatric, alcoholic, psychiatric, and patients with dentures [1, 2]. Types of FBs that could cause digestive tract injury due to their sharp ends include numerous types such as; fishbone, chicken bone, toothpick, Jujube pit, etc. [2]. The timing of presentation varies significantly, where the majority of presentations are late (>2 weeks), owing to the inability of the patient to recall swallowing the FB and the time of the FB to induce symptoms [3, 4]. The preferred investigation of choice is undetermined because most of the patients, if not all, will not recall ingesting a FB, or because of lack of clear history due to the patient’s factors; therefore, a formal workup of abdominal pain initially with an abdominal X-ray is required, which rarely detects the presence of FB, abdominal CT which has high sensitivity in detected FB if radiopaque, and no oral contrast to obscure the bowel lumen [5, 6]. Further, it can detect associated complications such as perforation, abscess, inflammatory process, etc. [3].

The most common sites of perforation account for the duodenum and the sigmoid colon; other sites include the esophagus, stomach, and small bowel [2]. The most common presentation is abdominal pain which accounts for 80%, nausea, vomiting, fever, and rarely shock due to sepsis [7]. Management depends on the patient clinical status, location of the FB, and presence of complications. When the patient has a confirmed diagnosis of FB ingestion, all of the above factors contribute to the decision-making [6].

An upper GI endoscopy is an effective choice for patients who report ingesting FB, and the diagnostic modalities locate it in the proximal GI tract, and the gastroenterologist shall determine accessibility [8]. The success rate of retrieval increases within the first 24 hours of ingestion [9, 10]. When an asymptomatic patient has a FB in the lumen of the distal GI tract that has not yet caused complication, a watchful waiting for the FB to pass with bowel motions is acceptable, provided that the FB is not a battery or magnet [3]. Surgical intervention is mandated when complications occur, and the approach should be determined based on the patient’s overall condition and the surgeon’s experience [9].

Diagnostic laparoscopy is feasible and effective in diagnosing and managing FB complications. Furthermore, it can be utilized to establish a diagnosis and treat the cause of the patient’s symptoms and any present complications when the diagnosis is in doubt [3].

## Conclusion

FB ingestion is a rare presentation; yet, serious complications could happen and be fatal, and a prompt intervention when complications occur is mandated to decrease the morbidity and mortality of the patient. A high index of suspicion, especially in the extremes of ages, and detailed history along with thorough investigation is crucial to reach a diagnosis. Endoscopy has a rule in the proximal GI tract FB; surgery whether laparoscopic or open is mandated in case of the presence of complications.

## Data Availability

All relevant data supporting the findings of this research are included within the manuscript or its supplementary materials.
